# Highly Efficient Light Absorption of Monolayer Graphene by Quasi-Bound State in the Continuum

**DOI:** 10.3390/nano11020484

**Published:** 2021-02-14

**Authors:** Tian Sang, Sina Abedini Dereshgi, Wisnu Hadibrata, Ibrahim Tanriover, Koray Aydin

**Affiliations:** 1 Department of Photoelectric Information Science and Engineering, School of Science, Jiangnan University, Wuxi 214122, China; 2 Department of Electrical and Computer Engineering, Northwestern University, Evanston, IL 60208, USA; sinaabedini2022@u.northwestern.edu (S.A.D.); wisnuhadibrata2022@u.northwestern.edu (W.H.); ibrahimtanriover2024@u.northwestern.edu (I.T.)

**Keywords:** light absorption, graphene, photonic crystal slab, quasi-bound state in the continuum

## Abstract

Graphene is an ideal ultrathin material for various optoelectronic devices, but poor light–graphene interaction limits its further applications particularly in the visible (Vis) to near-infrared (NIR) region. Despite tremendous efforts to improve light absorption in graphene, achieving highly efficient light absorption of monolayer graphene within a comparatively simple architecture is still urgently needed. Here, we demonstrate the interesting attribute of bound state in the continuum (BIC) for highly efficient light absorption of graphene by using a simple Si-based photonic crystal slab (PCS) with a slit. Near-perfect absorption of monolayer graphene can be realized due to high confinement of light and near-field enhancement in the Si-based PCS, where BIC turns into quasi-BIC due to the symmetry-breaking of the structure. Theoretical analysis based on the coupled mode theory (CMT) is proposed to evaluate the absorption performances of monolayer graphene integrated with the symmetry-broken PCS, which indicates that high absorption of graphene is feasible at critical coupling based on the destructive interference of transmission light. Moreover, the absorption spectra of the monolayer graphene are stable to the variations of the structural parameters, and the angular tolerances of classical incidence can be effectively improved via full conical incidence. By using the full conical incidence, the angular bandwidths for the peak absorptivity and for the central wavelength of graphene absorption can be enhanced more than five times and 2.92 times, respectively. When the Si-based PCS with graphene is used in refractive index sensors, excellent sensing performances with sensitivity of 604 nm/RIU and figure of merit (FoM) of 151 can be achieved.

## 1. Introduction

Graphene has attracted a high level of research interest due to its exceptional optical, electronical, chemical, and mechanical properties, combined with its unique electronic band structure [1,2]. For example, graphene has the linear dispersion of two-dimensional (2D) Dirac fermions, the full spectrum response from the ultraviolet to terahertz band, and the ultrahigh carrier mobility, revealing great potential for advancing modern optoelectronic devices [3,4]. In mid-infrared to terahertz (THz) spectral range, the absorption and confinement of light in graphene can be remarkably enhanced due to the excitation of graphene surface plasmon polaritons (GSPPs) [5]. However, in the visible (Vis) to near-infrared (NIR) wavelength region, graphene behaves mostly like an absorptive dielectric and cannot sustain GSPPs. It has been shown that the optical absorption of a suspended monolayer graphene only reaches a value of 2.3% under normal incidence in the Vis and NIR regions [6]. The poor light–graphene interaction constitutes a major limitation for its substantial applications in optoelectronic devices. Therefore, one of the challenges concerning this ultrathin material is to enhance its optical absorption efficiency.

To enhance the light absorption of graphene in Vis–NIR region, several mechanisms such as guided-mode resonance [7,8,9], coherent absorption [10], cavity resonance [11,12], plasmonic resonance [13,14], and prism coupling [15,16] have been proposed to enhance the light–graphene interaction. Unfortunately, the peak values of light absorption in monolayer graphene are confined in the range of 30–60% in most cases [9,10,11,12,13,14,15]. To significantly improve the light absorption of graphene, an effective approach is to integrate the graphene with the metasurfaces supported by the metallic mirror [17,18,19,20,21] or metallic structured substrate [22,23]. However, due to the inherent ohmic loss of metal materials, the use of the metallic mirror or structured substrate will inevitably result in suppression of light absorption in graphene. On the other hand, dielectric Bragg mirror does not have the issue of ohmic loss, and it has been reported that the monolayer graphene integrated with the all-dielectric Bragg mirror can achieve perfect or near-perfect light absorption [24,25,26,27,28,29]. In addition, the absorption of graphene can be efficiently improved by integrating graphene with a metal thin film and dielectric Bragg mirror via Tamm plasmon polaritons [30,31]. However, the use of the dielectric Bragg mirror increases the physical footprint of the device and makes the configuration more complicated, since Bragg multilayers consisting of high/low index materials require high-precision fabrication techniques.

In recent years, bound states in the continuum (BIC) have been demonstrated as an effective approach to enhance the interaction between light and matter, examples of present-day uses include directional lasing [32], magneto-optical phenomena [33], second-order nonlinear effects [34], giant enhancement of the Goos–Hänchen shift [35], ultrahigh–Q guided resonances [36] and much more. BIC also bring exciting opportunities to enhance light absorption of graphene due to the associated strong field enhancement. Zhang et al. [37] showed that optical absorption in graphene can be enhanced based on BIC using a sphere–graphene–slab structure, and ultrasensitive absorption of graphene can be obtained via the excitation of GSPPs. Later, Wang et al. [38] showed that light absorption in graphene can be controlled by asymmetric parameter of suspended silicon nanodisk, and maximum absorption of 50% can be realized at critical coupling through quasi-BIC in the two-port system composed of graphene. However, achieving highly efficient light absorption of monolayer graphene within a comparatively simple architecture is still rare, and a theoretical model based on critical coupling for perfect or near-perfect light absorption of graphene in the two-port system needs to be developed.

In this work, near-perfect absorption of the monolayer graphene is realized in the communication band by using the BIC based on the photonic crystal slab (PCS) of Si. The high confinement of light and near-field enhancement in the Si-based PCS with a slit contributes to the unexpected improvement of graphene absorption, where BIC turns into quasi-BIC due to the symmetry-breaking of the structure. Theoretical formulas based on the coupled mode theory (CMT) are derived that can well evaluate the absorption performances of graphene integrated with the symmetry-broken PCS. The absorption properties of the monolayer graphene are robust to the variation of the structural parameters. By using the full conical incidence, the angular bandwidths for peak absorptivity and the central wavelength of graphene absorption can be effectively improved. In addition, excellent sensing performances can be realized when the Si-based PCS with graphene is used in refractive index sensors.

## 2. Basic Principles and CMT for Absorption Enhancement of Graphene

**Figure 1:**
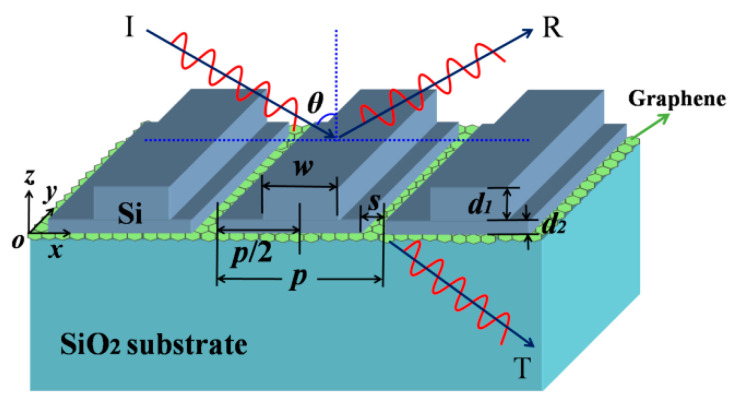
Schematic diagram of a subwavelength Si-based photonic crystal slab (PCS) with a monolayer graphene at the incident angle of *θ*. The monolayer graphene is sandwiched between the Si PCS and the SiO_2_ substrate, the structural symmetry of the PCS is broken due to the slit in the sublayer. *d*_1_ and *d*_2_ stand for the thickness of the grating layer and the sublayer, respectively. The width of the grating layer is *w*, and the width of the slit in the sublayer is *s*; the period is *p*.

The schematic image of the Si-based PCS with monolayer graphene is shown in Figure 1, where a monolayer graphene is sandwiched between a one-dimensional (1D) periodic Si PCS and a SiO_2_ substrate. The PCS consists of a grating layer and a sublayer, and the structural symmetry of the PCS is broken due to the narrow slit in the sublayer. The refractive index of Si is 3.48, the refractive index of SiO_2_ is 1.47, and the background is air. In practice, the Si-based PCS with monolayer graphene can be fabricated by combing the chemical vapor deposition (CVD) with nanolithography processes. Firstly, the monolayer graphene is directly deposited on the bare SiO_2_ substrate using the copper-vapor-assisted CVD technique [39]. Next, the Si layer is deposited on the top of the monolayer graphene by plasma-enhanced CVD [40]. Finally, the periodic Si PCS can be achieved by UV holographic lithography, reactive-ion etching with a CHF_3_ + SF_6_ gas mixture, and O_2_ashing for residual photoresist mask removal [41].

In the Vis–NIR region, graphene can be modeled as an ultrathin dielectric film with thickness of *t_g_* = 0.34 nm, and its relative permittivity can be written as *ε_g_* = 1 + *jσ_g_*/*ωε_0_t_g_*, where *σ_g_* represents the graphene surface conductivity, *ω* indicates the angular frequency, and *ε*_0_ is the vacuum permittivity. According to the Kubo formula, the graphene surface conductivity *σ_g_* can be written as the sum of the intraband *σ_intra_* and interband conductivity *σ_inter_* [42]:

(1) $$\sigma_{intra}(\omega )=-j\frac{e^{2}k_{B}T}{\pi \hbar^{2}(\omega -2j\Gamma )}[\frac{\mu_{c}}{k_{B}T}+2\ln(e^{-\frac{\mu_{c}}{k_{B}T}}+1)]$$

(2) $$\sigma_{inter}(\omega )=-j\frac{e^{2}}{4\pi \hbar }\ln[\frac{2\left|\mu_{c}\right|-(\omega -j2\Gamma )\hbar }{2\left|\mu_{c}\right|+(\omega -j2\Gamma )\hbar }]$$

where *ħ* and *e* are reduced Planck’s constant and the elementary charge, respectively. *μ_c_* is the chemical potential, *k_B_* is the Boltzmann constant, *τ* is the momentum relaxation time, and *Γ* = 1/2*τ* is the phenomenological scattering rate. The physical parameters of the graphene are set as *μ_c_* = 0.15 eV, T = 300 K, and *τ*= 0.5 ps.

**Figure 2:**
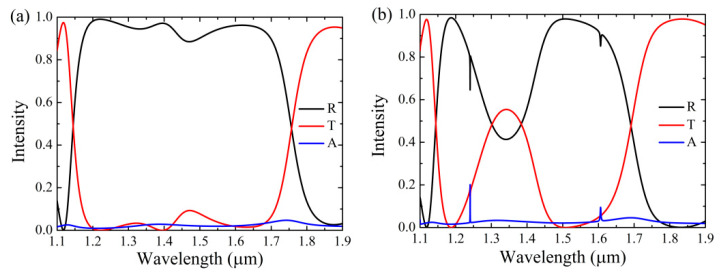
Spectral responses of the Si-based PCS under the TM-polarized normal incident wave. The parameters are: *p* = 690 nm, *d*_1_ = 438 nm, *d*_2_ = 249 nm, *w* = 345 nm. (**a**) *s* = 0. (**b**) *s* = 10 nm.

Figure 2 shows the spectral response of the Si-based PCS under the illumination of TM wave (magnetic field vector lies along the *y*-axis) at normal incidence (*θ* = 0°). Rigorous coupled-wave analysis (RCWA) [43] is used to calculate the spectral responses as well as other properties of the Si-based PCS. The frequency-dependent absorption of graphene is calculated as A = 1 – R− T, where R and T represent reflection and transmission, respectively. As can be seen in Figure 2a, the Si-based PCS with graphene shows broadband reflection with high reflectivity due to high index contrast of the grating [44]. In addition, the absorption response does not show spectral selectivity because of the wavelength-independent absorption of graphene in the NIR region. However, as can be seen in Figure 2b, a narrow slit in the sublayer with *s* = 10 nm will significantly alter the spectral responses due to the symmetry-breaking of the structure. In particular, the reflection spectrum is varied abruptly—with the occurrence of two resonant reflection dips, two narrow absorption peaks of graphene are formed at the locations of the two resonances as well. Obviously, the narrow slit in the sublayer is crucial for the selective absorption enhancement of the monolayer graphene.

**Figure 3:**
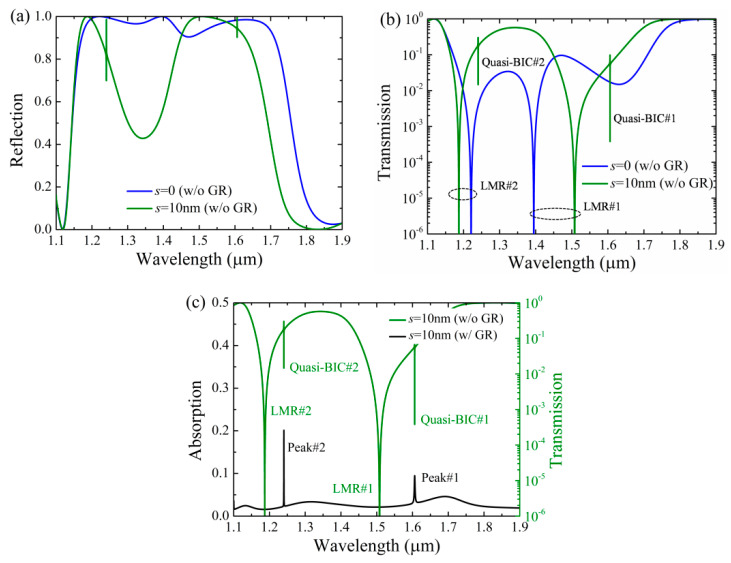
Spectral responses of the Si-based PCS without graphene (w/o GR) and with graphene (w/ GR). Other parameters are the same as those in Figure 2. (**a**) Reflection responses with *s* = 0 and *s* = 10 nm. (**b**) Transmission responses with *s* = 0 and *s* = 10 nm. (**c**) Absorption and transmission responses with *s* = 10 nm.

To corroborate the correlation of the location between the resonant reflection dips and the absorption peaks of the monolayer graphene, spectral responses of the Si-based PCS without graphene and with graphene are investigated. As can be seen in Figure 3a, for the Si-based PCS without graphene, the spectrum shows mirror effect as *s* = 0, while it varies abruptly and two Fano resonances are appeared as *s* is slightly altered (*s* = 10 nm). In both cases, the reflection responses are similar as in Figure 2, except for higher reflectivity because there is no absorption from graphene. In Figure 3b, transmission responses are in the logarithmic scale so as to clearly see the resonant locations of Si-based PCS without graphene. As can be seen in Figure 3b, there are two leaky mode resonances (LMRs) in the wavelength region of interest as *s* = 0; the LMRs arise from the coupling between the diffracted orders and the waveguide modes, and the overlapping of the two LMRs will result in broadband reflection with high reflectivity [45]. However, two additional high-Q Fano resonances occurred as *s* = 10 nm, where BIC turns into quasi-BIC due to the symmetry-breaking of the structure [46,47]. At normal incidence, the BIC mode can be completely decoupled from the radiation continuum due to its different symmetry, while it can be leaky by breaking the symmetry of the structure [48,49]. As can be seen in Figure 3c, the locations of the absorption peaks of the graphene are coincided with those of the quasi-BICs as there is no graphene in the structure, confirming that it is the quasi-BICs instead of the LMRs that are responsible for the absorption enhancement of the monolayer graphene.

**Figure 4:**
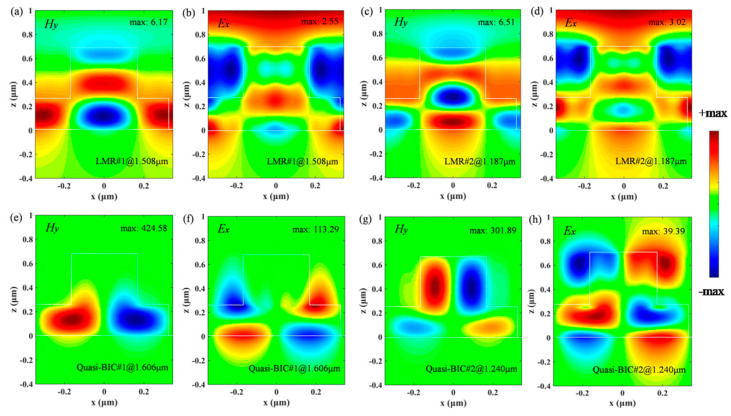
Distributions of electromagnetic fields associated with the leaky mode resonances (LMRs) and quasi-bound states in the continuum (BICs) of the Si-based PCS without graphene as *s* = 10 nm. Other parameters are the same as those in Figure 2. *H_y_* and *E_x_* denote the tangential components of the magnetic and electric field amplitudes, respectively, the values are normalized by incident field amplitudes. (**a**) *H_y_* and (**b**) *E_x_* of the LMR#1. (**c**) *H_y_* and (**d**) *E_x_* of the LMR#2. (**e**) *H_y_* and (**f**) *E_x_* of the quasi-BIC#1. (**g**) *H_y_* and (**h**) *E_x_* of the quasi-BIC#2.

To gain further insight into the physical basis for the absorption enhancement of the monolayer graphene, distributions of the tangential components (*H_y_* and *E_x_*) of electromagnetic fields associated with the LMRs and quasi-BICs of the Si-based PCS without graphene are shown in Figure 4. As can be seen in Figure 4a,b, the electromagnetic fields associated with the LMR#1 are slightly enhanced with the symmetrical mode (even mode) profiles to the *yz* plane. The magnetic field of the LMR#1 is mainly confined in the grating layer and the sublayer, and the moderate enhancement of the magnetic field with the maximum amplitude of 6.17 is responsible for large rejection bandwidth and low Q resonance of the Si-based PCS. In Figure 4c,d, it can be seen that the field distributions of the LMR#2 have similar properties as those of LMR#1. However, as can be seen in Figure 4e–h, in the case of the quasi-BIC modes, significant field enhancement can be realized with the anti-symmetrical mode (odd mode) profiles with respect to the *yz* plane, and the field distributions show the typical radiation patterns of electric dipole aligned with the *x*-axis. Leakage radiation to the surface normal direction is permitted due to the symmetry-breaking of the structure, which permits the overlap between the incident plane wave and the supported mode and consequently allows coupling between the fields [48]. Note the fields of the quasi-BIC#1 are mainly localized in the sublayer, while the fields of the quasi-BIC#2 are enhanced not only in the sublayer but also in the grating layer due to its high-order leaky mode features. In both cases of the quasi-BIC modes, although the intrinsic attenuation of graphene remains unchanged, the significant near-field enhancement and confinement of light in the Si-based PCS contributes to high Q Fano resonances as well as the unexpected improvement of graphene absorption.

**Figure 5:**
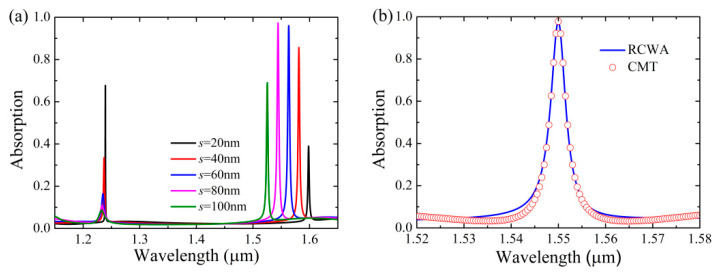
Absorption response of the Si-based PCS with graphene, other parameters are the same as Figure 2. (**a**) Absorption response as a function of the slit width *s*. (**b**) Rigorous coupled-wave analysis (RCWA) result and coupled mode theory (CMT) result of absorption response with *s* = 74 nm.

Figure 5a shows absorption response of the Si-based PCS with graphene as a function of the slit width *s*. As can be seen in Figure 5a, the absorption of graphene associated with the quasi-BIC modes can be flexibly controlled by merely varying the slit width, and the locations of the two absorption channels of graphene are blue-shifted as *s* is increased due to the decrease in the effective refractive index of the structure. In particular, the location and the peak absorptivity of the absorption channel of the quasi-BIC#1 (longer wavelength) can be effectively tuned by the slit width because its nearfields are mainly localized in the sublayer. By properly selecting the slit width as *s* = 74 nm with other structural parameters kept the same, near-perfect absorption of the monolayer graphene (97.8%) can be obtained at the wavelength of *λ_r_* = 1.55 μm with the full width at half maximum (FWHM) of Δ*λ* = 4.0 nm, as indicated by the theoretical RCWA result in Figure 5b. Compared with the previous graphene absorbers whose absorption efficiency is not high in the two-port system composed of graphene [8,9,10], and those metal mirror [17,18,19,20,21] or multilayer Bragg mirror [24,25,26,27,28,29] structuresthat are required to realize high absorption of graphene at critical coupling, the proposed absorber exhibits the advantage of achieving high absorption efficiency of graphene within a comparatively simple two-port system.

To further the quantitative understanding of highly efficient light absorption of the monolayer graphene in the Si-based PCS, theoretical formulas based the CMT are derived here. According to the CMT, the amplitude reflection (*ρ*) and transmission (*ξ*) coefficients of the grating-based 1D PCS can be written as [50,51]:

(3) $$\rho =r\frac{\left(\omega -\omega_{0})+i(\delta -\gamma \right)}{\left(\omega -\omega_{0})+i(\delta +\gamma \right)}$$

(4) $$\xi =t\frac{(\omega -\omega_{0})+i\gamma }{\left(\omega -\omega_{0})+i(\delta +\gamma \right)}$$

where *ω*_0_ is the resonant frequency; *r* and *t* are the Fresnel reflection and transmission coefficients of off-resonance, as no resonance is taking place in the structure. *γ* is the external leakage rate of the Si-based PCS, and *δ* is intrinsic loss rate due to the embedded monolayer graphene. The corresponding intensity of reflection (R) and transmission (T) can be expressed as:

(5) $$R=\rho \cdot \rho *=\frac{r^{2}[{\left(\omega -\omega_{0}\right)}^{2}+{\left(\gamma -\delta \right)}^{2}]}{{\left(\omega -\omega_{0}\right)}^{2}+{\left(\gamma +\delta \right)}^{2}}$$

(6) $$T=\xi \cdot \xi *=\frac{t^{2}[{\left(\omega -\omega_{0}\right)}^{2}+\gamma^{2}]}{{\left(\omega -\omega_{0}\right)}^{2}+{\left(\gamma +\delta \right)}^{2}}$$

Note for the Si-based PCS without graphene, the intrinsic loss rate *δ*=0 and *r*^2^ + *t*^2^ = 1 due to the subwavelength periodicity. In this case, R + T = 1 according to Equations (5) and (6). For the Si-based PCS with graphene, the absorption A = 1–R−T can be written as:

(7) $$A=\frac{(1-r^{2}-t^{2})[{\left(\omega -\omega_{0}\right)}^{2}+\gamma^{2}]+2(1+r^{2})\gamma \delta +(1-r^{2})\delta^{2}}{{\left(\omega -\omega_{0}\right)}^{2}+{\left(\gamma +\delta \right)}^{2}}$$

It can be inferred from Equation (7) that the absorption can be simplified as $A=1-\frac{r^{2}[{\left(\omega -\omega_{0}\right)}^{2}+{\left(\gamma -\delta \right)}^{2}]}{{\left(\omega -\omega_{0}\right)}^{2}+{\left(\gamma +\delta \right)}^{2}}=1-R$ if there is no transmission (*t*^2^ = 0). In addition, the absorption responses are in the form of the Lorentzian lineshape as $A=\frac{(1-r^{2}-t^{2})\gamma^{2}+2(1+r^{2})\gamma \delta +(1-r^{2})\delta^{2}}{{\left(\gamma +\delta \right)}^{2}}$ at the resonant frequency (*ω* = *ω*_0_). In particular, perfect absorption with A = 100% is realizable at critical coupling with *γ* = *δ* if *t*^2^ = 0, thus it is feasible to achieve highly efficient light absorption of graphene at critical coupling based on the destructive interference of transmission light within a comparatively simple structure composed of graphene. The absorption responses of the Si-based PCS with graphene are dependent on the off-resonance reflection (*r*) and transmission (*t*) coefficients, the external leakage rate (*γ*), and the loss rate (*δ*). Therefore, it is possible to achieve high absorption of graphene in the Si-based PCS by tuning the structural parameters.

To verify the validation of the theoretical model based on the CMT, the absorption response using the CMT is also shown in Figure 5b. In simulation, we extracted the total quality factor Q_tot_ of the Si-based PCS with graphene as Q_tot_ = *ω*_0_/Δ*ω* = *ω*_0_/2(*γ* + *δ*), where Δ*ω* is the FWHM at the resonant frequency *ω*_0_, thus we got *γ* + *δ* =1.57 × 10^12^ Hz as Q_tot_ = 387.5. The external leakage rate *γ* associated with the quasi-BIC mode can be obtained by using the Fano lineshape equation [52]:

(8) $$T_{\mathrm{Fano}}={\left|a_{1}+ja_{2}+\frac{b}{\omega -\omega_{0}+j\gamma }\right|}^{2}$$

to fit the transmission intensity spectra of the Si-based PCS without graphene, where *a*_1_, *a*_2_, and *b* are real number. The external leakage rate can be fitted as *γ* = 8.07×10^11^ Hz, and thus *δ* = 7.63 × 10^11^ Hz. As shown in Figure 5b, the absorption response of the CMT based on Equation (7) is in good agreement with that of the RCWA, validating that the proposed model could provide a general strategy to evaluate the absorption performance of graphene integrated with all-dielectric metasurface.

## 3. Performance Analysis and Sensing Application

**Figure 6:**
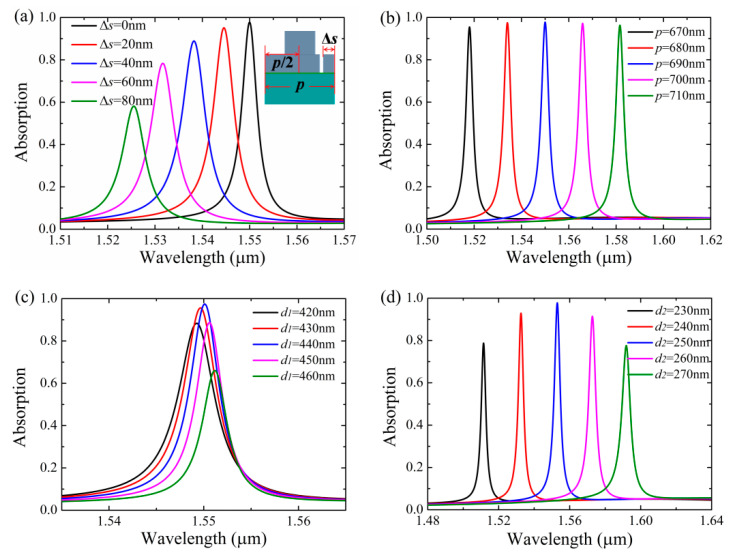
Absorption spectra of the Si-based PCS with graphene under the variations of structural parameters with *s* = 74 nm, other parameters are the same as Figure 2. (**a**) The lateral shift of the slit Δ*s* where Δ*s* is indicated in the figure inset. (**b**) The period *p*. (**c**) The thickness of the grating layer *d*_1_. (**d**) The thickness of the sublayer *d*_2_.

To evaluate the robustness of graphene absorption associated with the quasi-BIC, we firstly investigate the structural parameters (Δ*s*, *p*, *d*_1_, *d*_2_) on the absorption performances of the Si-based PCS with graphene. As can be seen in Figure 6a, as the slit is laterally shifted from the edge (Δ*s* = 0) to the center of the unit cell with *s* fixed as 74 nm, the location of the absorption peak is blue-shifted, and the peak absorptivity is reduced as well due to the reduced symmetry of the structure. In particular, as the slit approaches the center of the unit cell with Δ*s* = (*p* − *s*)/2 = 308 nm, the Si-based PCS becomes the symmetrical structure, and the absorption peak of graphene is vanished due to the symmetry incompatibility between the BIC mode and the external radiation. In Figure 6b, it can be seen that the location of the absorption peaks can be dynamically tuned with high absorptivity maintained by varying the grating period. In Figure 6c,d, it can be seen that high absorption enhancement of graphene can be achieved even the thickness of the grating layer and sublayer are significantly altered. The grating layer is related to the coupling strength between the diffracted order and the incident wave [45], thus the variation of *d*_1_ mainly changes the absorption bandwidth of the graphene. However, the variation of *d*_2_ mainly results in the shift of absorption peak of graphene because the mode profiles of the quasi-BIC#1 are mainly confined in the sublayer. Key parameters of the absorption spectra of the Si-based PCS with graphene are listed in Table 1, where *λ_r_*, A_peak_, and FWHM denote the absorption wavelength, the peak absorptivity, and the absorption bandwidth, respectively. Note the graphene absorption associated with the quasi-BIC mode is relatively stable to the variations of the structural parameters of Δ*s*, *p*, *d*_1_, and *d*_2_, which facilitates the robust control of absorption enhancement of graphene integrated with the Si-based PCS.

**Figure 7:**
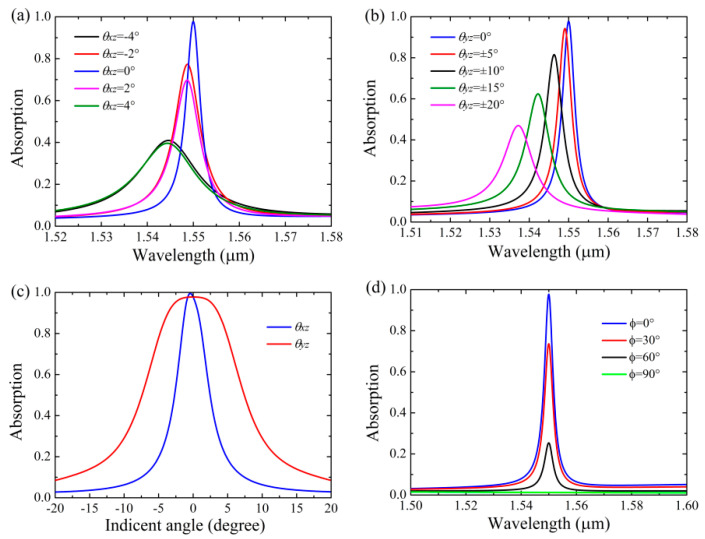
Absorption spectra of the Si-based PCS with graphene under the variations of incident angle *θ* and polarization angle *ϕ* with *s* = 74 nm, other parameters are the same as Figure 2. (**a**) Absorption spectra as a function of the incident angle in the *xz* plane. (**b**) Absorption spectra as a function of the incident angle in the *yz* plane. (**c**) Angular response for the central wavelength of 1.55 μm in both the *xz* and *yz* planes. (**d**) Absorption spectra as a function of the polarization angle.

We further characterized the incident angle and polarization angle dependence of the Si-based PCS with graphene. Figure 7a shows absorption spectra of the Si-based PCS with graphene at classical incidence, i.e., the incident angle (*θ_xz_*) is varied in the *xz* plane. As can be seen in Figure 7a, deviation from normal incidence will broaden the absorption bandwidth with the absorption peaks blue-shifted, and the peak absorptivity of graphene is reduced as well. The peak absorptivity of graphene is reduced to ~40.0% as *θ_xz_* = ±4°. Note because the Si-based PCS with a slit is anti-symmetric with respect to its mirror-symmetry plane (*yz* plane), the absorption spectra of graphene for the ±*θ_xz_* are slightly different. Figure 7b shows absorption spectra of the Si-based PCS with graphene at fully conical incidence, i.e., the incident angle (*θ_yz_*) is varied in the *yz* plane. It can be seen that the angular tolerance of graphene absorption can be effectively improved compared with that of the classical incidence, and the peak absorptivity of 47.0% can be achieved even at *θ_yz_* = ±20°. In this case, the absorption spectra for the ±*θ_yz_* are exactly the same because the incident angle is varied within the mirror-symmetry plane of the structure. Therefore, the angular tolerance for peak absorptivity of graphene at classical incidence can be enhanced more than five times by using the fully conical incidence. In general, the leaky modes of the grating-based metasurface are essentially related to the diffraction orders, and the angular variations of the *m*th-order diffracted wave vector for classic (|Δ*k*_diff_|_classic_) and full conic (|Δ*k*_diff_|_fully conic_) incidences can be written as [53]:

(9) $${\left|\Delta k_{\mathrm{diff}}\right|}_{\mathrm{classic}}=k_{0}\sin\theta$$

(10) $${\left|\Delta k_{\mathrm{diff}}\right|}_{\mathrm{fully}\mathrm{conic}}=\sqrt{{\left(k_{0}\sin\theta \right)}^{2}+{\left(2m\pi /p\right)}^{2}}-2m\pi /p$$

where *k*_0_ is the wave vector of the incident light, *θ* is the incident angle. In the subwavelength regime, (*p* < *λ*_0_), and $\left|\frac{p\sin\theta }{m\lambda_{0}}\right|<1$, thus Equation (10) can be rewritten in terms of |Δ*k*_diff_|_classic_ via Taylor series expansion as:

(11) $${\left|\Delta k_{\mathrm{diff}}\right|}_{\mathrm{full}\;\mathrm{conic}}=\frac{2m\pi }{p}\{\sqrt{{\left(\frac{p\sin\theta }{m\lambda_{0}}\right)}^{2}+1}-1\}\approx \frac{p\sin\theta }{2m\lambda_{0}}{\left|\Delta k_{\mathrm{diff}}\right|}_{\mathrm{classic}}$$

According to Equation (11), |Δ*k*_diff_|_fully conic_ is always smaller than |Δ*k*_diff_|_classic_, thus the absorption spectra at fully conical incidence is more robust than that at classical incidence as the incident angle is varied.

Figure 7c shows the angular response for the central wavelength of 1.55 μm in both classical (*θ_xz_*) and fully conical (*θ_yz_*) incidences, and the structural parameters are the same in both two cases. As shown in Figure 7c, the angular bandwidth of absorption at classical incidence is 5.1°, while it can be further enhanced 2.92 times to 14.9° by using the fully conical incidence, and the results are in line with the prediction of Equation (11). Notably, perfect absorption of graphene can be achieved as *θ_xz_* is slightly deviated from normal incidence due to the decreased symmetry of the structure. Compared with the previous Si-based graphene absorbers that can achieve high absorption at 1.55 μm only at oblique incidence within a small angle range of ±1.5° [54,55], the proposed absorber can achieve high absorption at normal incidence due to the symmetry-breaking of the structure, and its angular tolerance can be significantly larger by using the fully conical incidence. Figure 7d shows absorption spectra of graphene as a function of the polarization angle, where *ϕ* = 0° and *ϕ* = 90° correspond to the TM and TE polarizations (electric field vector lies along the *y*-axis), respectively. As can be seen in Figure 7d, as the polarization angle is varied from 0° to 90°, the peak absorptivity of graphene at the central wavelength of 1.55 μm can be dynamically tuned in the range between 97.8% and 1.2% with the low sideband level kept almost the same, which may be used to fashion the functions of notch filters and intensity-resolved sensors based on thin-film gratings or photonic crystals.

**Figure 8:**
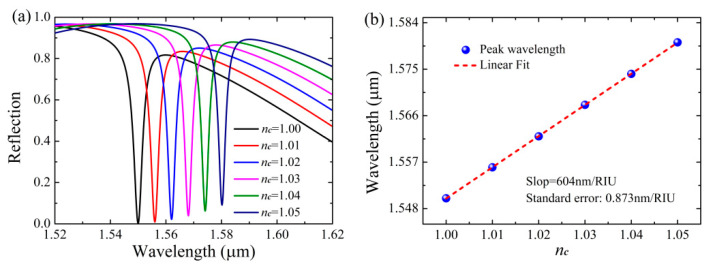
Sensing properties of the Si-based PCS with graphene, other parameters are the same as Figure 2 except *s* = 74 nm. (**a**) Reflection spectra as a function of the refractive index of the background. (**b**) Locations of the reflection dip as a function of the refractive index of the background.

Finally, we showed that the Si-based PCS can function as a high sensitivity refractive index sensor due to the robust absorption of the monolayer graphene. Here, we evaluate the refractive sensing capabilities of the Si-based PCS with sensitivity S = Δ*λ*_peak_/Δ*n_c_* and figure of merit (FoM) = *S*/Δ*λ*, where Δ*λ*_peak_ is the peak wavelength change with the refractive index change Δ*n_c_*. and Δ*λ* is the half-width of the absorption band. As can be seen in Figure 8a, the reflection spectra of the Si-based PCS with graphene are very sensitive to the variation of the background refractive index *n_c_*. The reflection dip of Si-based PCS with graphene is red-shifted with the increase in *n_c_*, but the reflectivity of the reflection dip is slightly increased due to deviation from the critical coupling for perfect light absorption of graphene. In Figure 8b, the slope of the curve via linear fitting shows that the sensitivity of Si-based PCS with graphene reaches 604 nm/RIU, and its FoM can be obtained as 151. In the NIR wavelength region, the sensitivity and FoM of the Si-based PCS with graphene are larger than many nanostructured absorbers such as Si bar-ring resonator [56], Si nanoblocks [57], plasmonic nanoslit-microcavity [58], and guided-mode resonance grating [59]. Additionally, the sensing performances of the Si-based PCS with graphene are comparable with the plasmonic gold mushroom, whose FoM is comparable to the theoretical upper limit for standard propagating surface plasmon resonance sensors [60]. Therefore, the Si-based PCS with graphene may be suitable for sensing applications due to the relatively simple architecture and excellent performances.

## 4. Summary

We provide a novel approach to achieve highly efficient light absorption of monolayer graphene via quasi-BIC by employing a compact Si-based PCS with a slit. Near-perfect absorption of the monolayer graphene can be realized due to the high confinement of light and near-field enhancement in the Si-based PCS, where BIC turns into quasi-BIC due to the symmetry-breaking of the structure. The derived formulas based on the CMT can well evaluate the absorption performances of graphene integrated with the symmetry-broken PCS, indicating that high absorption efficiency of graphene is feasible at critical coupling based on the destructive interference of transmission light within a comparatively simple two-port system. The excitation of the quasi-BIC of the Si-based PCS is robust to the variation of the structural parameters such as the lateral shift of the slit, the period, the thickness of the grating layer and the sublayer, and high absorption of graphene can be maintained even the structural parameters are significantly altered. Moreover, the angular tolerance of graphene absorption for the classical incidence can be effectively enhanced by using the full conical incidence, the angular bandwidths for the peak absorptivity and for the central wavelength of 1.55 μm can be enhanced more than five times and 2.92 times, respectively. When the Si-based PCS with graphene is used in refractive index sensors, excellent sensing performances with sensitivity of 604 nm/RIU and FoM of 151 can be realized. The proposed architecture together with its design principle reveals the potential implementation of BIC for extraordinary absorption enhancement of monolayer graphene, which may provide a guide to design graphene and other 2D material-based optoelectronics devices.

## Figures and Tables

**Table 1:** Key parameters of the absorption spectra of the Si-based PCS with graphene shown in Figure 6.

Parameters	Δ*s* (nm)			*p* (nm)			*d*_1_ (nm)			*d*_2_ (nm)		
	0	40	80	670	690	710	420	440	460	230	250	270
*λ_r_*(nm)	1550	1538	1526	1518	1550	1582	1549	1550	1551	1512	1550	1592
A_peak_	97.8%	88.9%	58.1%	95.5%	97.8%	96.3%	88.3%	97.5%	66.0%	78.8%	97.7%	77.6%
FWHM (nm)	4.0	6.1	6.2	3.7	4.0	4.2	4.9	3.9	3.4	3.3	4.0	6.2
